# NKX2-1 New Mutation Associated With Myoclonus, Dystonia, and Pituitary Involvement

**DOI:** 10.3389/fgene.2018.00335

**Published:** 2018-08-22

**Authors:** Péter Balicza, Zoltán Grosz, Viktor Molnár, Anett Illés, Dora Csabán, Andras Gézsi, Lívia Dézsi, Dénes Zádori, László Vécsei, Mária Judit Molnár

**Affiliations:** ^1^Institute of Genomic Medicine and Rare Disorders, Semmelweis University, Budapest, Hungary; ^2^Department of Neurology, Faculty of General Medicine, Albert Szent-Györgyi Clinical Centre, Univesity of Szeged, Szeged, Hungary; ^3^MTA-SZTE Neuroscience Research Group, Szeged, Hungary

**Keywords:** *NKX2-1* gene, *NKX2-1* related disorders, benign hereditary chorea, brain-lung-thyroid syndrome, chorea, myoclonus dystonia, pituitary, empty sella

## Abstract

**Background:** *NKX2-1* related disorders (also known as brain-lung-thyroid syndrome or benign hereditary chorea 1) are associated with a wide spectrum of symptoms. The core features are various movement disorders, characteristically chorea, less frequently myoclonus, dystonia, ataxia; thyroid disease; and lung involvement. The full triad is present in 50% of affected individuals. Numerous additional symptoms may be associated, although many of these were reported only in single cases. Pituitary dysfunction was ambiguously linked to *NKX2-1* haploinsufficiency previously.

**Case Presentation:** We examined two members of a family with motor developmental delay, mixed movement disorder (myoclonus, dystonia and chorea) and endocrinological abnormalities (peripheric thyroid disease, and pituitary hormone deficiencies). Dystonia predominated at the father, and myoclonus at the daughter. The father had hypogonadotropic hypogonadism, while the daughter was treated with growth hormone deficiency. Both patients had empty sella on MRI. Candidate gene analyses were negative. Exome sequencing detected a pathogenic stop variation (NM_003317:c.338G>A, p.Trp113^*^) in the *NKX2-1* gene.

**Conclusions:** This case study has two highlights. (1) It draws attention to possible pituitary dysfunction in brain-lung-thyroid syndrome, and provide further evidences that this might be linked to loss of function of the *NKX2-1* gene. (2) It underscores the importance of considering *NKX2-1* related disorders in the differential diagnosis of myoclonus dystonia.

## Introduction

*NKX2-1* (*TTF1*) related disorders are associated with a wide spectrum of symptoms, which may be present in various combinations. The most frequently reported symptoms are related to alterations in the central nervous system, thyroid or lung tissues, hence the name brain-lung-thyroid disorder (Peall and Kurian, 2015). Brain involvement may manifest among other things as motor developmental delay, hypotonia, chorea, myoclonus, ataxia, drop attacks, and psychiatric disorders. The neurologic syndrome is also known as benign hereditary chorea (OMIM 118700) because the disease is typically non-progressive. Thyroid involvement manifests as congenital hypothyroidism, or thyroid agenesis, while lung involvement may be present as infant respiratory distress syndrome, recurrent pulmonary infections, and interstitial lung disease. Besides the core features, many other associated features were described such as malignancies, short stature, skeletal abnormalities, hypoparathyroidism (Peall and Kurian, 2015). One case was reported in the literature, where growth hormone (GH) deficiency was documented in association with a whole gene deletion (Peall et al., 2014b). However, whether this was incidental or could be linked to the *NKX2-1* mutation was equivocal.

## Patients and methods

**Figure 1:**
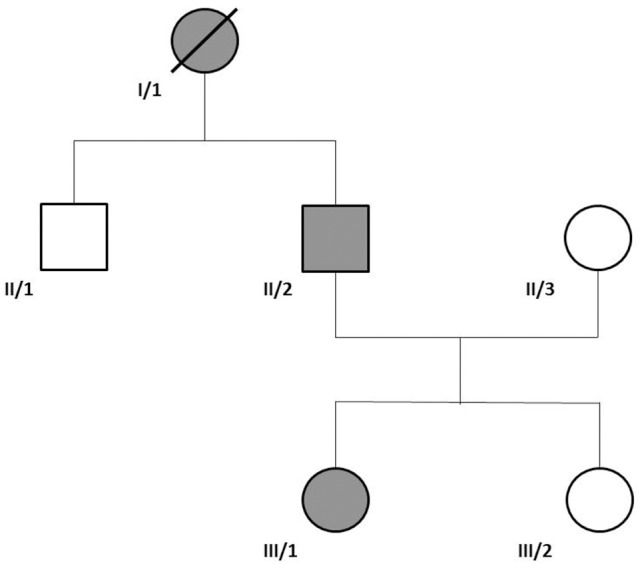
Family tree of the affected family. We have examined patient II/2 and III/1. Family history suggested autosomal dominant inheritance.

Here we present the case of two affected members of a family (**Patient II/2** and **III/1** on Figure 1), who—besides the motor phenotype- show evidence for the involvement of the pituitary, associated with an *NKX2*-1 point mutation. This article is a retrospective case study; it has an institutional ethical committee approval. Informed consent for diagnostic genetic testing was obtained from each individual. The patients gave informed, written consent for publication of the history, examinations, and videos.

For exome sequencing genomic DNA library preparation was performed by using Agilent SureSelectQXT Human All Exon v5 reagents (Agilent Technologies, Santa Clara, CA, USA) according to the manufacturer's protocol. Library preparation was followed by next-generation sequencing using Illumina HiSeq PE Cluster Kit v4 for cluster generation on cBot and HiSeq SBS Kit v4 for sequencing on HiSeq2500 system (Illumina, San Diego, CA, USA). Filtering of variants was based on the inheritance pattern, allele frequency, and consequence of the mutation. Pathogenicity of the variants was assessed as recommended by the American College of Medical Genetics guideline 2015 (Richards et al., 2015).

## Case presentation

### Figure 1: family tree

**Figure 2:**
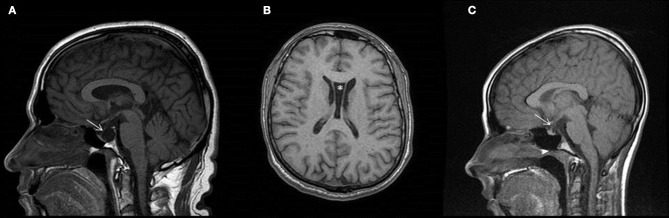
Brain MR images of the examined patients. T1 weighted images of Patient II/2 **(A,B)**, and of Patient III/1 **(C)**. Image **(A)** and **(C)** demonstrate empty sella on sagittal images. Infundibulum sign is identifiable (arrows), thus proving empty sella vs. cystic mass of pituitary.

**Patient II/2** (46 years old at the time of exome sequencing) presented with motor developmental delay. He started to walk at age 2 years, but the gait was always unsteady. At the age of 6 years, child neurologist described ataxic gait, with appendicular dyskinesias, which were mainly noticeable during the walk. During elementary school, hyperkinetic movements were increasing, but not disabling for the patient. During high school years, perioral hyperkinesis also developed. Later the coordination problems and hyperkinetic symptoms slowly progressed, and after the age of 35 years, he was not able to work anymore as a tailor. Besides hyperkinetic movements, cervical dystonia also developed, later bothersome myocloni appeared in the diaphragm. At the age of 45 years a low testosterone and low luteinizing hormone (LH) level was detected in the background of erectile dysfunction. He received subcutaneous human chorionic gonadotropin (hCG), and later intramuscular testosterone-undecanoate treatment. Elevated thyroid-stimulating hormone (TSH) level was also detected at the patient, he underwent levothyroxine supplementation. Because of asthma the patient was treated by a pulmonologist as well. Repeated brain MRIs showed cave of septum pellucidum and empty sella (Figure 2). Abdominal ultrasound showed diffuse increased echogenicity of the liver, corresponding to hepatic steatosis. Earlier candidate gene examinations (SCA1,2,3,6,7,17 repeat expansion analysis, IT15 repeat analysis, POLG, DYT1 gene sequencing) were negative. At the time of the exome sequencing, hyperkinetic movements were already treated with tetrabenazine, and the patient regularly received botulinum toxin for his cervical dystonia. Supplementary Video S1 shows elements of the neurological examination. We detected mild perioral hyperkinesis, dysarthria, mild (treated) cervical dystonia. Myocloni were present in the abdominal musculature, and mild choreiform movements in the extremities, fingers, and trunk, which were increasing during walk. Mild spasticity and dystonia was noticed in the lower extremities, but pyramidal signs were not present. In Romberg position, mild unsteadiness was present, but nor gait neither appendicular ataxia was detected. His mother (**I/1**) had similar movement difficulties as described by the patient, but we could not examine her, because she died earlier due to colon cancer.

His daughter (**Patient III/1**, 20 years old at the time of the exome sequencing) also experienced motor developmental delay. She started to walk around age of 1.5 year, but she was unsteady. Gait ataxia, muscle hypotony, and myoclonic hyperkineses were described at that time by a pediatric neurologist. Hyperkineses were noticed at age 4 years, falls occurred at age 5 years. Later, the intensity of hyperkineses increased. Because of low stature, insulin tolerance test with measurement of growth hormone (GH) level was performed at age of 13 years. No GH elevation was detected (GH remained under 10 ng/mL). Brain MRI showed an empty sella (Figure 2). She received subcutaneous GH between the age of 13–17 years, and she reached a normal height. TSH level was normal. During elementary school, the performance was always somewhat below the average. An educational psychologist diagnosed dyslexia, with normal IQ. Later at age of 18 years, marginal IQ was measured (83 points in the Wechsler test 83). She started high-school education, but was unable to keep up with lectures. Around the age of 18 years, psychiatric symptoms emerged with exaggerated anxiety, depression and suicidal temptation. She was treated with a transient psychotic episode after an induced abortion, because of unwanted pregnancy. Former candidate genetic studies were negative (SCA1,2,3 repeat expansion, GNAL, RELN, DYT11, DYT5, DYT14 sequencing). Elements of the neurologic examination at age of 20 years are shown on the Supplementary Video S2. Mild microcephaly, underdeveloped secondary sex characteristics, and joint hypermobility were noticed. Continuous choreiform movements were detected in the head, trunk and extremities, with superimposed myocloni. Dysarthria was noticeable as well. Mild spasticity and Babinski sign was detected in the right lower limb. Ataxia was not present.

### Figure 2: brain MRIs of the patients

Myoclonus dystonia was suspected first at the family, given the pronounced myoclonus at the daughter and predominant dystonic features at the father, but targeted *SGCE* sequencing was negative. Exome sequencing was then performed at the two patients. A pathogenic heterozygous stop variant was detected in both patients in the *NKX2-1* gene: NM_003317:c.338G>A, p.Trp113^*^. This variant was not present in the ExAc, 1000G, ClinVar, dbSNP databases, nor in our exome cohort of 200 individuals. Pathogenic variants of the *NKX2-1* gene are associated with the brain-lung-thyroid syndrome, which is compatible with the phenotype of the patients. After the diagnosis, TSH screening was performed at the daughter, with normal results. Chest X-ray was normal in both patients.

## Discussion and conclusions

While retrospectively most of the symptoms are consistent with the literature, this case study has two highlights. Firstly, it draws attention to possible pituitary dysfunction in brain-lung-thyroid syndrome, and provide further evidences that this might be linked to loss of function of the *NKX2-1* gene. Secondly, it underscores the importance of considering *NKX2-1* related disorders in the differential diagnosis of myoclonus dystonia.

Both of our patients showed evidence of pituitary dysfunction. The father had low LH levels, leading to hypogonadism, while the daughter had low GH levels, causing her low stature. Besides, the pituitary of both patients was structurally also affected, as they had empty sella. Only scarce information is available in the literature about pituitary dysfunction in humans in brain lung thyroid syndrome. Current evidences suggest that probably loss of function of *NKX2-1* gene associates with the pituitary abnormalities, however this must be further investigated by functional studies. In the cohort of Peall et al. (Peall et al., 2014b), one patient with a whole *NKX2-1* gene deletion had growth hormone deficiency. Salvatore et al. reported a family with congenital hypothyroidism, where two patients had empty sella on brain MRI associating with a stop mutation of *NKX2-1* gene (Salvatore et al., 2010). However no information is available about the pituitary hormones in this family. Accornero et al. reported a patient with duplication of the pituitary stalk and subclinical hypothyroidism, who had a heterozygous 1.2 Mb deletion containing the *NKX2-1, MBIP, NKX2-8, PAX-9, and SLC25A1* gene (Accornero et al., 2010). This patient showed an exaggerated response of prolactine to TRH, but otherwise the hypothalamus-pituitary-gonadal, -somatotropic and -adrenal axis function was normal. One family, with a nonsense variant, reported by Veneziano et al., showed evidence of cystic pituitary masses (Veneziano et al., 2014). This patient had primary hypothyroidism, but the remainder of the pituitary hormonal profile was normal.

From the study of *NKX2-1* (*TTF-1*) in the mouse, it is known, that it has important roles in the developing brain. Indeed, it is responsible for the interneuron specification of medial ganglionic eminence cells (Butt et al., 2008), and the regulation of the direction of the migrating interneurons (Nóbrega-Pereira et al., 2008). *TTF1* knockout mice lack the pituitary gland (Takuma et al., 1998). Postnatally it regulates GH and prolactin transcription in the rat pituitary (Lee et al., 2007) and has also been shown to play a role in the regulation of circadian changes in gonadotropin-releasing hormone (GnRH) expression (Matagne et al., 2012). Therefore, *NKX2-1* haploinsufficiency is a plausible explanation for the symptoms of our patients. Our case report serves as further evidence for the involvement of both pituitary development, and function in *NKX2-1* related disorders in humans. However, this aspect of the syndrome is needed to be further studied in a cohort of patients.

Another important note to this case is the necessity of remembering *NKX2-1* gene in the differential diagnosis of myoclonus dystonia. In our case, the predominant dystonia in the father and frequent myocloni in the daughter led probably to the targeted testing of other movement disorder associated genes. Our first hypothesis focused on myoclonus dystonia, but *SGCE* testing was negative. It is known from the literature that *NKX2-1* related disorders may resemble myoclonus-dystonia. However certain features may help the clinical diagnosis, such as aggravation of myoclonic jerks with movements in *SGCE* positive patients, and continuous chorea in *NKX2-1* positive patients (Asmus et al., 2007). In contrast with this observation, at the studied family the father's hyperkinesis worsened with movements, especially during gait. On the other hand, the predominant motor phenotype was dystonia at him. The daughter showed myoclonic jerks, superimposed on continuous chorea. In the study of Peall et al. (2014a), from 70 suspected myoclonus-dystonia patients, negative for *SGCE* mutations, two cases were found to have putative *NKX2-1* mutations, and no mutations were detected in the *DYT1, GCH1, THAP1* genes. In addition to these genes, *ADCY5* need also be considered in SGCE negative myoclonus dystonia (Chang et al., 2016). We have non-systemically checked commercially available dystonia panels (Supplementary Table 1) whether *NKX2-1* was represented on them. We also checked NCBI Genetic Testing Registry (https://www.ncbi.nlm.nih.gov/gtr/) and looked specifically for *NKX2-1* gene (geneid: 7080) whether the testing of this gene is present as a member of a dystonia panel. We found only one from 11 dystonia panels, on which this gene was present. Thus it is important to bear in mind the *NKX2-1* gene as a possibility in hyperkinetic syndromes featuring any variety of dystonia, myoclonic jerks, and chorea.

Both of our patients received tetrabenazine for the treatment of chorea. The daughter had more severe phenotype, she received 12.5 mg tetrabenazine three times a day. This reduced choreiform movements, but significant chorea still remained. We could not give larger doses because of sedation, and depression in the history. As the patient had also pronounced gait impairment we also tried levodopa, but she not tolerated even small doses (levodopa/benserazide 50 mg/12.5 mg three times a day). She also received small dose of levetiracetam (2 × 12.5 mg), which had positive effect on myocloni. Depression and anxiety was treated with escitalopram and alprazolam effectively. The fathers choreiform movements were well controlled with 2 × 12.5 mg tetrabenazine, without side effects. Cervical dystonia was treated effectively with botulinum toxin injections.

## Data availability statement

The datasets used and/or analyzed during the current study are available from the corresponding author on reasonable request.

## Author contributions

PB, ZG, DZ, LD, and MM examined patients, summarized clinical data, and wrote the article. VM and PB performed next generation sequencing data analysis, AG supporting bioinformatics. AI and DC performed genetic laboratory works. MM and LV coordinated the work and reviewed the manuscript. All authors read and approved the final manuscript.

### Conflict of interest statement

The authors declare that the research was conducted in the absence of any commercial or financial relationships that could be construed as a potential conflict of interest.

## Supplementary material

Supplementary Video S1 Neurological examination of patient II/2.

Supplementary Video S2 Neurological examination of patient III/1.

Supplementary Table 1 Genes represented on commercial dystonia panels.
